# A Longitudinal Study of Stress in New Nurses in their First Year of Employment

**DOI:** 10.1155/2022/6932850

**Published:** 2022-11-21

**Authors:** Yan Fang, Jingping Yang, Mengling Zhang, Jihong Song, Rongjin Lin

**Affiliations:** ^1^Nursing Department, The First Affiliated Hospital of Fujian Medical University, Fuzhou 350005, Fujian Province, China; ^2^School of Nursing, Fujian Medical University, Fuzhou 350122, Fujian Province, China

## Abstract

**Objective:**

This study aimed to analyze changes in occupational stress in new nurses during the first year of employment.

**Methods:**

A prospective longitudinal study was conducted from 2020 to 2021 using one questionnaire four times on 127 newly employed nurses in a tertiary general teaching hospital in the province of Fujian.

**Results:**

The results showed that new nurses had moderate to high levels of stress in all four stages, with the highest stress level at 4 and 8 months of employment and the lowest stress level at 12 months; the differences in stress scores at different time points were statistically significant (*p* < 0.05). The trends in each stressor dimension varied across different periods. The highest scores were for pressure caused by “time allocation and workload,” which peaked in month 8. The same trend was observed for stress from “patient care” and “work environment and equipment.” “Management and interpersonal relationships” scored the highest overall stress score at the start of employment before declining. The lowest stress score was from “work environment and equipment” at the start of employment, and the lowest was from “management and interpersonal relationships” from month 4 onward.

**Conclusion:**

New nurses had higher overall occupational stress during their first year of employment under different stressors. Therefore, nursing managers should actively focus on stress factors of new nurses and provide targeted interventions to help them during their training period.

## 1. Introduction

Nursing is a stressful occupation, with stress stemming from high expectations, excessive responsibility, and minimal empowerment [1]. Excessive occupational stress may be detrimental to an individual's physical and mental health, negatively impacting productivity and increasing the likelihood of caregivers leaving by reducing their job satisfaction [2, 3]. In 2019, a previous study showed that the attrition rate in nursing was 11.3%, nearly twice that of physicians (6%), and nursing positions were among the most difficult to fill in healthcare [4].

Newly employed nurses are transitioning from study to work. As new nurses are only beginning to apply their theoretical knowledge to clinical practice, they are not yet able to meet the demands of their new work environment. They also lack communication skills, teamwork experience, and the practical knowledge of more experienced nurses. These factors, coupled with interpersonal challenges from frequent departmental rotations, mean that new nurses face various transitional shocks and heavy mental and psychological pressures, and they are more likely to leave their jobs [5]. According to a survey, 75% of new nurses leave due to high stress levels, and 83.75% believe there is a lack of humanistic care [6]. One study investigated new nurses' desires to leave at different times and found that 17.2% wanted to leave at the beginning of their employment; this reached 59.6%, 74.4%, and 71.8% at 4, 8, and 12 months after employment, respectively [7], illustrating how the desire to leave changes over time. In 2017, the Institute of Hospital Management of the National Health Commission analyzed the original nurse turnover data voluntarily reported by 940 hospitals, covering 30 provinces on the national nursing quality data platform, and concluded that the nurse turnover rate in China was 2.15% [8]. During the pandemic, a large number of medical staff members rushed to the front line of antiepidemic fight. In order to strengthen the epidemic prevention and control work, hospitals also invested more human resources in epidemic prevention posts, such as nucleic acid sampling, fever clinics, and ward attendant control, which aggravated the shortage of nurses' human resources, thus shortening the standardized training cycle of new nurses and forcing them to work independently in advance. All of which increased the pressure on new nurses and became the possible reason for their resignation. Barnes believes that standardizing training for 6 months to 2 years after initial employment could help student nurses transition smoothly to the workplace [9], while Dillon et al. highlight the importance of a successful transition to encourage nurses to stay in the workforce [10]. Therefore, this longitudinal study aimed to examine how stress changed in the nurses' first year of employment and analyze the trends of those changes and the main stressors, which may provide evidence-based, comprehensive, and dynamic interventions to help nurses in their transition period.

## 2. Participants and Methods

### 2.1. Participants

A convenience sampling method was used. Newly employed nurses, who were not employed previously in a tertiary grade A general teaching hospital in the province of Fujian, were selected as survey participants in this prospective study. The inclusion criteria were as follows: nurses in their first year of employment, those who volunteered to be part of this study, and those who provided informed consent. The exclusion criteria were as follows: nurses who were previously employed, nonrecent graduates, and those who resigned during the study period.

According to the rough estimation formula of the sample size, the sample size *N* = [Max (the number of scale dimensions) × (15∼20)] × [1 + (15%∼20%)]. There are five dimensions in the “Nurse Stressor Scale in China,” and the minimum calculated sample size was 120.

### 2.2. Methods

#### 2.2.1. Tools

The Chinese version of the Nurse Stressor Scale [11] was used to assess work-related stress. It includes 35 questions and is scored on a 4-point scale. It is a multidimensional self-report scale that generates assessment scores across five dimensions, including nursing profession and work-related problems, time allocation and workload problems, work environment and equipment problems, patient care problems, and management and interpersonal relationship problems. The scores range from 35 to 140, and higher scores indicate a higher level of work stress. In the present study, Cronbach's alpha coefficient of five domains ranged from 0.83 to 0.95, suggesting good internal consistency.

Sociodemographic data include gender, education, department, number of night shifts, household registration, and residence status.

#### 2.2.2. Data Collection

**Figure 1:**
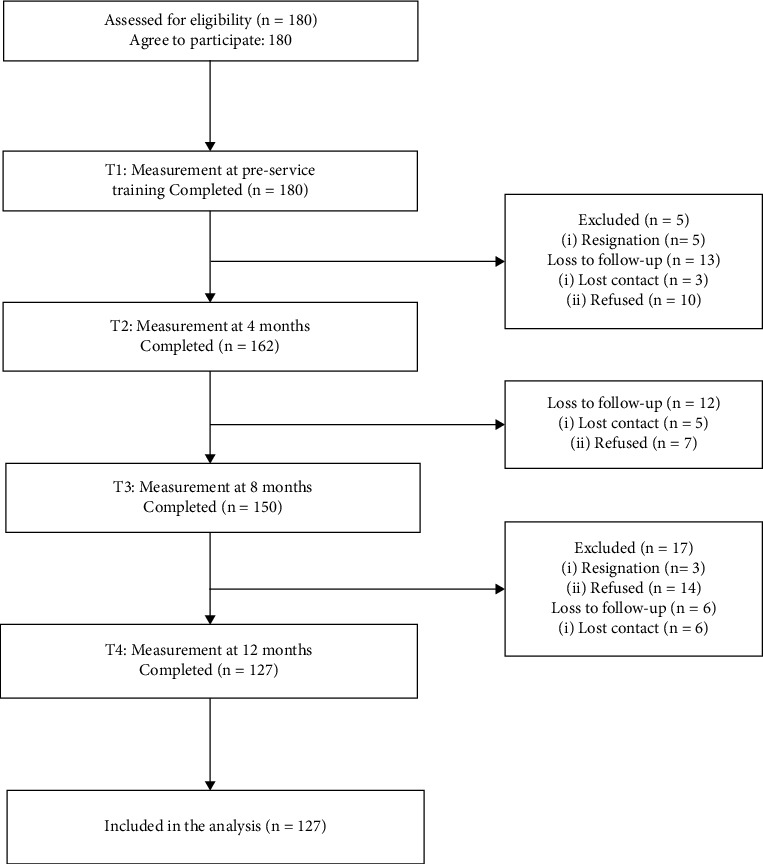
The recruitment process and the flow of the study.

Questionnaires were distributed at preservice training (T1) and at 4 (T2), 8 (T3), and 12 (T4) months after entry. The T1 questionnaires were given to participants after they had given informed consent following their preservice training. Preservice training is designed to help new nurses acknowledge basic clinical skills, medical laws, and theoretical knowledge, which are beneficial to adapting clinical work for new nurses. The research team members explained questionnaires to participants, distributed questionnaires directly, collected them on-site, and established a WeChat group to facilitate the survey. The data on questionnaires were collected from July 2020 to July 2021. The participants were followed up for 12 months. The recruitment process and the flow of the study are presented in Figure 1.

#### 2.2.3. Statistical Analyses

Data were analyzed using IBM SPSS Statistics version 26.0. Descriptive analyses were conducted for demographical and all dimensions of the Nurse Stressor Scale. One-way repeated-measures analysis of variance (ANOVA) was used to assess changes in the nurse's work-related stress across the investigation. The Bonferroni post hoc test was used to examine differences between the mean scores on stress from T1 to T4. If Mauchly's spherical hypothesis held (*p* > 0.05), the results were analyzed using an uncorrected coefficient. However, if it did not (*p* < 0.05), the results were analyzed using the Greenhouse–Geisser correction coefficient. A two-sided test was used, and *p* < 0.05 was considered statistically significant.

#### 2.2.4. Quality Control

To improve survey response rates, questionnaires were distributed, collected, and reviewed by the research team. The team also ensured that participants understood the content and completed the questionnaire. The inclusion criteria were followed to ensure data accuracy, and a WeChat group was established to communicate with participants. For the questionnaires that were completed on-site, any missing items were checked and added at the time of collection. Questionnaires were completed online using the Questionnaire Star tool, and omissions were avoided by setting required fields. Data were inputted and checked by two people to avoid errors.

#### 2.2.5. Ethical Considerations

For the protection of privacy, an agreement was signed, informing participants that the survey was for research purposes only and that their personal information would not be divulged. Informed consent was obtained by explaining the purpose of the study to participants. A guarantee to respect the participants' rights was given, and they were told that their participation was voluntary.

## 3. Results

### 3.1. General

Of the 127 participants, 116 (91.3%) were female, 37 (29.1%) had a bachelor's degree or above, and 90 (70.9%) had a junior college degree. A total of 116 (91.3%) were registered in the province, and 11 (8.7%) were registered outside. Finally, 104 (81.9%) lived in a rented department, and 23 (18.1%) were homeowners.

### 3.2. Changes in the Participants' Stress Scores

**Figure 2:**
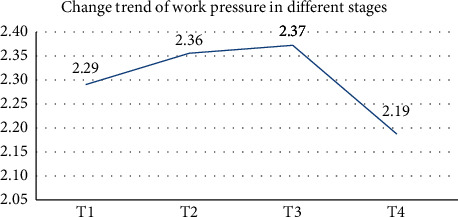
Trends of job stress of the survey respondents in different stages.

The results from the repeated-measures ANOVA are shown in Table 1. They showed that Mauchly's sphericity test (*p* < 0.05) (*W* = 0.294) did not hold, and the model corrected by the Greenhouse–Geisser method showed statistically significant differences among stress scores (*p* < 0.05) (*F* = 74.789). Pairwise comparisons showed that the differences between T1 and T2, T1 and T3, T1 and T4, T2 and T4, and T3 and T4 were statistically significant (*p* < 0.05 in all groups). However, the difference between T2 and T3 was not statistically significant (*p* > 0.05). The stress score trends at different times were trapezoidal (see Figure 2). The highest and lowest stress levels were at 8 and 12 months, respectively, after commencing employment.

### 3.3. Changes in the Participants' Stressor Scores

**Figure 3:**
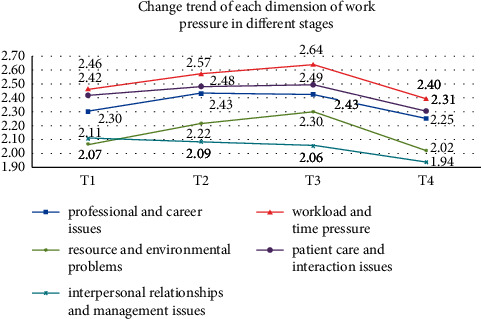
Trends of job stress in each dimension of the survey respondents in different stages.

The results from the repeated-measures ANOVA of the five job stress dimensions are shown in Table 1. They revealed that the changes in each stressor score were statistically significant (*p* < 0.05), and the trends are shown in Figure 3. The greatest stressor in all four stages was “time allocation and workload,” which peaked at T3. Stress from “patient care” and “work environment and equipment” also peaked at T3. “Management and interpersonal relationships” scored the highest overall stress score at T1 and then declined. The lowest stress score was from “work environment and equipment” at T1, while the lowest was from “management and interpersonal relationships” from T2 onward.

## 4. Discussion

### 4.1. Changes in Stress Scores

This study showed that stress in all four stages was moderate to high, which was higher than the findings of Lou [12]. Occupational stress levels were significantly higher at months 4 and 8 than those at preemployment and month 12, and differences were statistically significant. Occupational stress at months 4 and 8 was similar. Occupational stress was the lowest at month 12, and differences were statistically significant. These results were inconsistent with those of Zhang et al. [13].

Studies have shown that the shorter the employment period of new nurses, the poorer their psychological capacity, which may be because of the varying stress loads at different stages [14]. During preservice training, new nurses lack subjective experience of the different duties of nurses. Moreover, as new nurses are about to enter a clinic, they will have career expectations. They will also be uncertain about their new department's team atmosphere, interpersonal relationships, and knowledge structure. Therefore, although stress could be intense at this time, they will still have hope.

After entering clinical practice, new nurses' work environments and social roles change and problems such as a lack of professional knowledge and poor adaptability can arise. In addition, affected by the major stress event of COVID-19, nurses recruited in 2020 stopped clinical practice in the middle of their internship and changed to online learning, so they lack hands-on ability and the ability to cope with the pandemic, which makes new nurses suffer different degrees of mental health problems [15]. According to Duchscher's transition theory, during their first 3 to 4 months of employment, new nurses experience the process of learning and adaptation [16], in which they must overcome the conflict between theory and reality and adapt to their new role. If there is insufficient adaptation time, this causes a great deal of stress, seriously affecting their growth and development.

It was considered that the high stress level at month 8 could be due to departmental rotation. Most hospitals in China arrange their rotation times according to the nature of the department based on the national syllabus recommendation, which is generally 3–6 months [17]. In this study, the second rotation was staggered at 6–7 months due to human resource problems. Month 8 was the adaptation period for a new department. New nurses had to adapt to a new working pattern and interpersonal relationships when they had already adapted to their original department, which resulted in new anxiety and uncertainty. Many studies have shown that new nurses have to rotate through multiple departments, and this working pattern makes it difficult to integrate into the departmental team, which could lead to negative psychological effects and reduce the efficacy of departmental training [13, 18, 19].

After 1 year of standardized training, new nurses significantly improved their theoretical knowledge, specialized operational skills, communication skills, and emergency and contingency abilities. They adapted to the rhythm of clinical work and developed core professional competencies. In addition, since they had been in their departments for 1 year, they cooperated well with their colleagues and had a stronger sense of belonging. Therefore, pressure was significantly reduced.

### 4.2. Different Stressor Trends

#### 4.2.1. Time Allocation and Workload

“Time allocation and workload” were the biggest stressors for new nurses in their first year (see Figure 3). Most training and assessments were arranged during departmental rotations in the standardized training program. Departments also arranged more training and assessments for new nurses than for ordinary nurses to teach them to be independent as quickly as possible. The nature of the shift system in nursing meant that their rest times often conflicted with training, and training after a day of high-intensity clinical practice made many new nurses feel physically exhausted. As examination results are linked to performance and training, the psychological pressure on new nurses increased, especially when rotated at month 8. As they had already passed their probationary examinations, they could fulfill the job independently. However, they were working in new departments and facing new types of disease and interpersonal relationships. Due to a lack of human resources, the head nurse sometimes let new nurses work independently. At this time, although new nurses might be unskilled, they often worked overtime and night shifts. A shortage of human resources and an imbalance between work and the nurses' private lives could cause some new nurses to burn out, leading to poor quality work and increased stress [20].

#### 4.2.2. Management and Interpersonal Relationships

At the beginning of their employment, new nurses were worried that their theoretical and operational skills would not meet the demands of the position or the needs of patients; similar results were found in a study by Sun et al. [21]. However, the present study found that the stress from “management and interpersonal relationships” was low. This could be because most participants had interned in the hospital and were familiar with each other and other nurses in the department. Therefore, loneliness was less of a problem, and the head nurse was attentive.

However, nurses often have to face consultations with patients and families, questions from the head nurse, and unexpected resuscitations in clinical practice. New nurses are not competent in these areas and worry that they would be unable to handle the situation effectively, becoming a burden to the team, being rejected by colleagues, and endangering patients. Therefore, the pressure on new nurses in this area would be medium. With accumulated clinical experience and repeated training and assessment, nurses become more confident, and the trust from patients, families, and colleagues increases. Nursing is a job that requires teamwork, and if colleagues work closely, interpersonal relationships can be more harmonious, leading to a decline in stress.

This study has some limitations. It was limited to a tertiary hospital, and the duration period was 1 year. Future studies should examine other levels of hospitals to assess the nurses' second year of employment. This would better depict stress trends, deepen the understanding of stress effects, and help new nurses better complete their role change and adapt quickly to clinical practice.

## 5. Conclusion

Dynamic changes in stress of new nurses in the first year were observed, with significantly higher job stress at months 4 and 8 after starting employment than at pre-entry and month 12 of employment. The highest scores in all dimensions of stress were for “time allocation and workload.” Nursing managers and new nurses should take varying measures to relieve stress in different periods and different dimensions.

This study suggests that nursing managers should be aware of the stress that new nurses face at different times and provide timely, effective, and continuous support. They should also be aware of new nurses' career plans to improve their sense of value and achievement. New nurses should adjust their psychological state, seek outside help, and work together during the transition period.

## Figures and Tables

**Table 1:** Comparison of the stress of the survey respondents in different stages.

Items	T1	T2	T3	T4	*F*	*p*	Post hoc
Total stress score	2.29 ± 0.36	2.36 ± 0.50	2.37 ± 0.48	2.19 ± 0.54	74.789	<0.001	T4 < T1, T2, T3 T1 < T2, T3 T2 = T3
Nursing professional and work-related problems	2.30 ± 0.41	2.43 ± 0.58	2.43 ± 0.53	2.25 ± 0.61	12.238	<0.001	T1 < T2, T3 T4 < T2, T3 T2 = T3, T1 = T4
Time allocation and workload problems	2.46 ± 0.49	2.57 ± 0.62	2.64 ± 0.64	2.40 ± 0.71	14.392	<0.001	T4 < T2, T3 T1 < T3 T1 = T2, T1 = T4 T2 = T3
Work environment and equipment problems	2.07 ± 0.44	2.22 ± 0.58	2.30 ± 0.59	2.02 ± 0.55	16.930	<0.001	T1 < T2, T3 T4 < T2, T3 T1 = T4, T2 = T3
Patient care-related problems	2.42 ± 0.49	2.48 ± 0.57	2.49 ± 0.59	2.31 ± 0.62	13.813	<0.001	T4 < T1, T2, T3 T1 = T2 = T3
Management and interpersonal relationship problems	2.11 ± 0.42	2.09 ± 0.57	2.06 ± 0.53	1.94 ± 0.51	8.532	<0.001	T4 < T1, T2, T3 T1 = T2 = T3

*Note*. T1: before employment; T2: at month 4 after employment; T3: at month 8 after employment; T4: at month 12 after employment *t*; *p* < 0.05.

## Data Availability

The datasets used and/or analyzed during the current study available from the corresponding author on reasonable request.
